# Enhanced Levels of Combined Waterlogging and Heat Stress Exacerbate Anatomical Damage, Photosynthetic Impairment, and Oxidative Stress in Tomato

**DOI:** 10.3390/antiox15091210

**Published:** 2026-09-20

**Authors:** Wen Qin, Yankai Li, Yang Sun, Rosa M. Rivero, Xiaoming Song, Ron Mittler, Fangling Jiang, Zhen Wu, Dong Xiao, Xuedong Yang, Rong Zhou

**Affiliations:** 1 Sanya Research Institute, College of Horticulture, Nanjing Agricultural University, Nanjing 210095, China; 2024804359@stu.njau.edu.cn (W.Q.); 2023204044@stu.njau.edu.cn (Y.L.); 2024104071@stu.njau.edu.cn (Y.S.); jfl@njau.edu.cn (F.J.); zpzx@njau.edu.cn (Z.W.); dong.xiao@njau.edu.cn (D.X.); 2 Center of Edaphology and Applied Biology of Segura (CEBAS-CSIC), Department of Plant Nutrition, Campus Universitario Espinardo, Ed. 25, 30100 Murcia, Spain; rmrivero@cebas.csic.es; 3 School of Life Sciences, North China University of Science and Technology, Tangshan 063000, China; songxm@ncst.edu.cn; 4 Division of Plant Science and Technology, College of Agriculture, Food and Natural Resources, Bond Life Sciences Center, University of Missouri, Columbia, MO 65201, USA; mittlerr@missouri.edu; 5 Advanced Agriculture Initiative, Tel-Hai University of Kiryat Shmona in the Galilee, Kiryat Shmona 1220800, Israel; 6 Horticulture Research Institute, Shanghai Academy of Agricultural Sciences, Shanghai 201403, China

**Keywords:** tomato, combined waterlogging and heat stress, leaf anatomy, stomata, photosynthesis, ROS homeostasis, proteostasis, photoprotection, heat shock response

## Abstract

Global climate change increases concurrent waterlogging and heat waves, threatening crop production. Tomato, a widely cultivated vegetable crop, is susceptible to both stresses, but their combined effects on tomato responses remain poorly understood. This study analyzed the anatomical, physiological, and molecular responses of two tomato genotypes, ‘NT212’ and ‘NT606’ (sensitive and tolerant to combined waterlogging and heat stresses), under control (C), waterlogging (W), heat stress (H1, H2, and H3) (34/27 °C, 38/31 °C, 42/35 °C), and combined stress (WH1, WH2, and WH3) for 48 h. ‘NT212’ exhibited only abaxial epidermal peeling under H2, but severe anatomical degradation including abaxial epidermal peeling and sponge parenchyma cell lysis under WH2. The superoxide anion (O_2_^•−^) production rate and *PsbS* and *HSP70* expression were significantly higher in ‘NT212’ under WH2 than W and H2. Moreover, as the stress intensity increased from WH1 to WH2 and WH3, the O_2_^•−^ production rate and *PsbS* and *HSP70* expression were significantly increased in ‘NT212’. In contrast, ‘NT606’ did not exhibit obvious cell lysis under any stress treatments. The O_2_^•−^ production rate was highest in ‘NT606’ under WH3 among all the treatments. *HSP70* expression was significantly upregulated in ‘NT606’ under H3 and WH3 compared with the other treatments. Consequently, ‘NT212’ exhibited severe wilting under the H2, H3, and all combined stresses, while ‘NT606’ showed severe wilting only under WH3. Thus, combined stress caused synergistic damage exceeding individual stress, with injury intensifying as temperature increased. This study provided vital knowledge for breeding climate-resilient tomato cultivars, especially under combined waterlogging and heat stress.

## 1. Introduction

Global climate change is characterized by an increased frequency and intensity of extreme weather events [1]. Concurrent abiotic stresses, such as waterlogging and heatwaves, are becoming common, posing a severe and complex challenge to agricultural productivity worldwide [2]. Unlike single stresses, combined stresses often trigger unique, non-additive physiological and molecular responses that cannot be extrapolated from studying each stress in isolation [3,4,5]. This interaction can be antagonistic, additive, or synergistic, with the latter representing the most damaging scenario where the combined effect exceeds the sum of individual stresses [3,6]. Understanding the impacts of combined stress on the growth of crops is therefore critical for developing climate-resilient crops.

Waterlogging depletes soil oxygen, leads to root hypoxia, disrupts root respiration and nutrient uptake, and results in the accumulation of toxic metabolites like ethanol and lactic acid [7,8,9]. For instance, barley shoot physiology is impaired by waterlogging stress, manifesting as stomatal closure, reduced photosynthetic rate, and leaf wilting [10]. Heat stress, on the other hand, directly affects protein stability, membrane fluidity, and the efficiency of the photosynthetic apparatus [11]. Key symptoms include thylakoid membrane damage, Rubisco deactivation, and enhanced photorespiration [12,13]. The plant’s primary defense involves the rapid activation of heat shock transcription factors (HSFs), which orchestrate the synthesis of molecular chaperones like heat shock proteins (HSPs), including heat shock protein 70 (HSP70), in order to maintain proteostasis [14,15,16].

When waterlogging and heat co-occur, their individual damaging effects likely converge and amplify [17]. Hypoxic roots under heat stress may experience accelerated metabolic dysfunction, while the shoot, simultaneously battling heat-induced water loss and waterlogging-induced hydraulic failure, faces a severe dilemma [17,18]. This can lead to a synergistic collapse of leaf anatomy, photosynthesis, and antioxidant systems. Leaf anatomy, a fundamental determinant of photosynthetic capacity and stress resilience, is highly sensitive to environmental cues [19,20]. Palisade and spongy parenchyma structures are optimized for light interception and gas diffusion; their disintegration under stress directly limits CO_2_ conductance and light absorption [21,22]. Stomata, the gatekeepers of gas exchange, show dynamic responses to both soil water status and aerial vapor pressure deficit (VPD), which is elevated under heat [23,24]. The interaction of root hypoxia (closing signal) and high VPD (closing signal) under combined stress may force stomata into extreme and potentially damaging closure states [25,26]. As stomata are typically closed in plants experiencing waterlogging stress, while heat stress requires stomata to be open for leaf cooling via transpiration, a conflict that may be similar to that experienced by plants during a combination of drought (leading to stomatal closer) and heat stress may also occur during a combination of waterlogging and heat stress [3,27,28].

Heat shock transcription factor A1 (*HSFA1*) serves as a master regulator orchestrating the initial plant response to heat stress [29,30], while *HSP70* functions as a crucial molecular chaperone essential for preventing protein aggregation and maintaining cellular homeostasis under thermal stress [15,31]. Despite their well-established protective roles and status as classic heat markers under single stressors, their dynamic expression patterns and functional necessity under the complex conditions of combined waterlogging and heat stress remain largely uncharacterized [18,32]. Moreover, Photosystem II subunit S (*PsbS*), a key protein in non-photochemical quenching (NPQ) that dissipates excess light energy as heat, is vital under stress conditions that promote light over-excitation [33]. Whether its regulation is sustained or overwhelmed under stress could determine the extent of photoinhibitory damage [34].

Tomato (*Solanum lycopersicum* L.), a globally important vegetable crop, is particularly susceptible to both waterlogging and heat stress applied individually [17,35,36]. Research on their combination in tomatoes, especially dissecting the integrated anatomical, physiological, and molecular cascades, remains however scarce. Therefore, this study aimed to decipher the synergistic damage induced by combined waterlogging and three levels of heat stress in tomato. Using two genotypes with contrasting susceptibilities being treated under individual and combined stress, this study systematically analyzed the changes in leaf anatomy and stomatal morphology, identified physiological responses (photosynthetic pigment content and gas exchange, reactive oxygen species/ROS accumulation), and compared the expression levels of key stress-responsive genes. Our study provides comprehensive phenotyping knowledge for enhancing the resilience of tomato plants to compound waterlogging and heat stress.

## 2. Materials and Methods

### 2.1. Plant Materials, Treatments, and Sampling

Based on our previous screen of multiple tomato genotypes, ‘NT212’ and ‘NT606’ from the Vegetable Physiology and Ecology Laboratory, Nanjing Agricultural University were selected to represent sensitive and tolerant genotypes to combined heat (38 °C) and waterlogging stress, respectively. Tomato seeds of the two genotypes were sown in 50-hole trays (54 cm length, 28 cm width) filled with substrate mixed with peat, vermiculite, and perlite (volume ratio = 2:1:1). Seedlings were cultivated in a greenhouse under a photoperiod of 14 h/10 h (day/night) with 300 μmol m^−2^ s^−1^ photosynthetic photon flux density (PPFD). The seedlings were irrigated with 1000 mL water per tray every five days. Ten days after sowing, the seedlings with one fully expanded leaf were irrigated with 1000 mL Stanley macronutrient water-soluble fertilizer (N + P_2_O_5_ + K_2_O ≥ 60%, nitrate nitrogen ≥ 5%, B = 0.08%, EDTA-Zn = 0.08%, EDTA-Fe = 0.08%) every four days. Seedlings with two fully expanded leaves were transplanted into pots (10 cm diameter, 8 cm depth) containing the same mixture of peat, vermiculite, and perlite at 16 days after sowing. Afterwards, each pot was irrigated with 100 mL of the same Stanley macronutrient water-soluble fertilizer (Stanley Agriculture Group Co., Linyi, China) every two days.

The 26-day-old seedlings were moved to climate chambers for two days of acclimation. Seedlings with four fully expanded leaves were divided into eight groups for the following eight treatments: control (C, 25/18 °C), waterlogging stress (W), heat stress (H1, 34/27 °C; H2, 38/31 °C; H3, 42/35 °C), and combined waterlogging and heat stress (WH1, waterlogging + 34/27 °C; WH2, waterlogging + 38/31 °C; WH3, waterlogging + 42/35 °C). The pots with the plants for the W, WH1, WH2, and WH3 treatments were placed into plastic containers (52 cm length, 27 cm width, 12 cm height) being filled with water according to the method of Zhou et al. [37]. The relative humidity was maintained at 50–60%, and the PPFD was 300 μmol m^−2^ s^−1^ (14 h/10 h, day/night) in the climate chambers. In total, 192 plants were studied with 12 plants per genotype per treatment. After 48 h of stress exposure, three randomly selected plants were evaluated for plant height and biomass and three randomly selected plants were applied for the observation of leaf anatomy and stomatal characteristics and the measurements of leaf relative chlorophyll content and photosynthetic parameters per genotype per treatment. The remaining six plants were collected as three biological replicates (with two plants for one biological replicate) for the remaining biochemical and molecular analyses. The second and third fully-expanded leaves of the plants were chosen for the measurements.

### 2.2. Observation of Leaf Anatomy and Stomatal Morphology

Sample preparation for leaf structure analysis followed the methodology of Lima et al. [20]. Leaf samples were fixed and stored in FAA solution (volume ratio of 70% ethanol, formaldehyde, and glacial acetic acid = 18:1:1). Dehydration was performed using an ascending ethanol gradient from 50% to 100%. Thereafter, leaf segments were cleared in xylene and embedded in paraffin following standard infiltration. Sections of 8 µm thickness were prepared using a rotary microtome (RM2235, Leica Biosystems, Nussloch, Germany), stained with safranin and fast green, which were observed using an upright fluorescence microscope with a 10 × objective lens (DM6 B, Leica Microsystems, Wetzlar, Germany).

Leaf stomatal anatomy was assessed using silicone rubber imprinting techniques [38]. Imprints were obtained from the second fully expanded leaf using impression material (Elite HD+, Zhermack, Badia Polesine, Italy). Afterwards, the imprint was transferred onto a microscope slide using nail polish. The samples were observed under an upright fluorescence microscope with a 63 × objective lens (DM6 B, Leica Microsystems, Wetzlar, Germany). For each biological replicate, three photos were taken, and three stomata were randomly chosen for measuring stomatal traits, including stomatal and pore length, width, and area (*n* = 27 per treatment).

### 2.3. Leaf Chlorophyll Content

Leaf relative chlorophyll content was measured using SPAD-502 chlorophyll meter (Konica Minolta, Tokyo, Japan). Three spots were taken per leaf, with the average value of the three spots as the relative chlorophyll content for each leaf. Leaf chlorophyll (0.1 g) was extracted using 10 mL 95% ethanol for 24 h in the darkness. With 95% ethanol as the blank, the chlorophyll content was measured at 649 nm and 665 nm using a microplate reader (Cytation3, BioTek, Winooski, VT, USA).

### 2.4. Leaf Photosynthetic Parameters

Net photosynthetic rate (P_n_), stomatal conductance (G_s_), intercellular CO_2_ concentration (C_i_), transpiration rate (T_r_), and leaf temperature of the third fully-expanded leaves were measured using a Li-6400 portable photosynthesis meter (LI-COR, Lincoln, NE, USA). Measurement parameters were set as follows: 1000 μmol m^−2^ s^−1^ light intensity, 500 μmol s^−1^ flow rate, 400 μmol L^−1^ CO_2_ concentration, and 50–60% relative humidity. Temperature of leaf cuvette was set to 25 °C for all treatments.

### 2.5. Leaf O_2_^•−^ Production Rate and H_2_O_2_ Concentration

Leaf O_2_^•−^ production rate was measured following the method of Zhou et al. [37]. The leaf samples (0.2 g) were placed in a mortar and ground into a homogenate with pre-cooled sodium phosphate buffer (0.05 M, pH 7.8). The homogenate was centrifuged at 4 °C and 13,800× *g* for 20 min. The samples were mixed with phosphate-buffered saline (0.05 M, pH 7.8) and 10 mM hydroxylamine hydrochloride solution. The mixture was incubated at 25 °C for 20 min and then mixed with aminobenzenesulfonic acid and α-naphthylamine, then incubated at 25 °C for another 20 min. After centrifugation at 900× *g* for 10 min, the absorbance of the samples was measured at 530 nm using a microplate reader (Cytation3, Bio Tek, Winooski, VT, USA).

Leaf H_2_O_2_ concentration was determined using the KI spectrophotometric method according to Pehlivan and Guler [39]. Leaf samples (0.1 g) were ground in a mortar with liquid nitrogen and then the samples were mixed with pre-chilled 0.1% trichloroacetic acid (TCA) solution. The homogenate was centrifuged at 13,800× *g* for 15 min and the supernatant was then collected. The samples were mixed with 1 M KI solution and phosphate buffer (0.1 M, pH 7.0) for 1 h in darkness. The absorbance of samples was measured at 390 nm using a microplate reader (Cytation3, BioTek, Winooski, VT, USA), with 0.1% TCA as the reference.

### 2.6. qRT-PCR (Quantitative Real-Time PCR)

Total RNA was extracted using RL lysate (AF504A, Proteinssci, Shanghai, China). The RNA was reverse transcribed using the All-in-One First-Strand Synthesis MasterMix kit (Yugong, Lianyungang, China), according to the manufacturer’s instructions. The reactions were performed using a reverse transcription system (Eppendorf, Hamburg, Germany). The qRT-PCR was performed using TOROGreen qPCR Master Mix kit (Toroivd, Shanghai, China). Reactions were performed in a QuantStudio^®^ 3 real-time PCR system (Thermo Fisher Scientific, Singapore). Primer sequences were designed using Primer 3 (Primer-E Ltd., Plymouth, UK) and shown in Table S1. Tomato *ACTIN* gene (Solyc03g078400) was used as the internal reference. The 10 µL reaction system included 5 µL TOROGreen qPCR Master Mix, 1 µL cDNA (at 5-fold dilution), 0.4 µL upstream and downstream primers and 3.2 µL ddH_2_O. Reaction procedure was as follows: 95 °C pre-denaturation for 60 s, followed by 40 cycles of 10 s at 95 °C and 30 s at 60 °C. Melting curve analysis was performed using the default parameters (5 s at 95 °C and 1 min at 65 °C).

### 2.7. Plant Height and Biomass

Plant height was measured using a ruler (accuracy 0.1 cm). Fresh weights of root, stem, and leaf were separately measured using an electronic balance. The root, stem, and leaf samples were then placed in an oven at 105 °C for 15 min, followed by drying at 65 °C for 48 h until constant weight was achieved. The stable value of dry matter was recorded as the dry weight.

### 2.8. Statistical Analysis

Leaf anatomy and stomatal structure parameters were quantified using ImageJ 1.53a (NIH, Bethesda, MD, USA). The relative expression of genes was calculated by using the 2^−ΔΔCt^ method. The one-way analysis of variance (ANOVA) was applied using IBM SPSS Statistics 26 (SPSS Inc. Chicago, IL, USA) according to Duncan’s test (*p* < 0.05). Figures were made using Microsoft Excel 2019 (Microsoft Corp., Redmond, WA, USA).

Interactions were analyzed using a general linear model (GLM) including the main effects and interaction terms of cultivar and treatment. Hierarchical clustering analysis based on squared Euclidian distance on the parameter matrix was conducted.

## 3. Results

### 3.1. Leaf Anatomy

**Figure 1:**
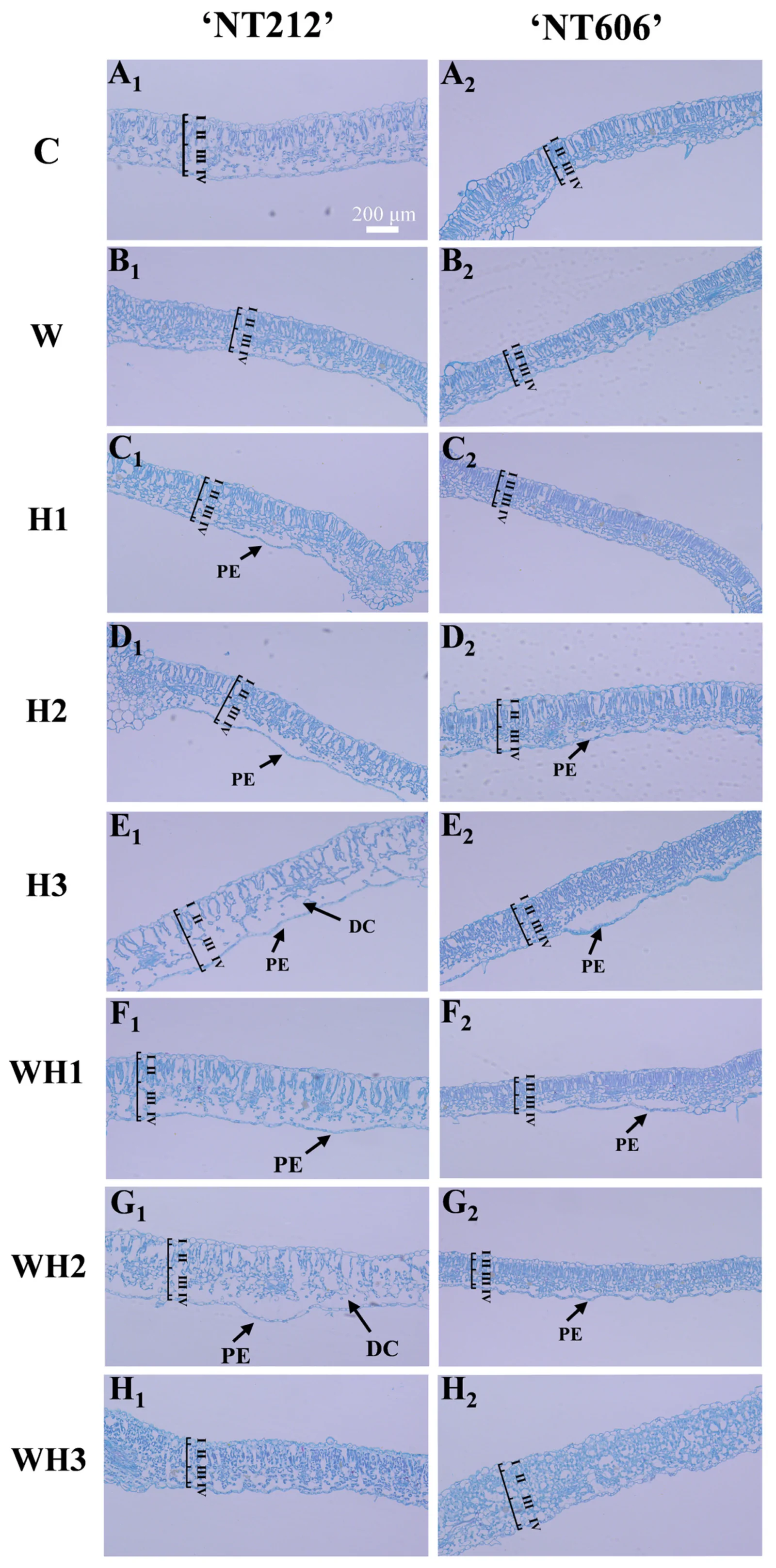
Leaf anatomy of ‘NT212’ and ‘NT606’ at (**A_1_**,**A_2_**) control (C, 25/18 °C), (**B_1_**,**B_2_**) waterlogging (W), (**C_1_**–**E_2_**) heat stress (H1, 34/27 °C; H2, 38/31 °C; H3, 42/35 °C), (**F_1_**–**H_2_**) combined waterlogging and heat stress (WH1, waterlogging + 34/27 °C; WH2, waterlogging + 38/31 °C; WH3, waterlogging + 42/35 °C) for 48 h. Note: I, II, III, and IV are the adaxial epidermis, palisade parenchyma, spongy parenchyma, and abaxial epidermis of tomato leaves, respectively. PE and DC indicate peeled epidermis and disintegrated cells. The scale bar in (**A_1_**) represents 100 μm, which can be applied for the rest of the subfigures. Observations were made at 100 × magnification.

A typical arrangement of the epidermis, palisade parenchyma, and spongy parenchyma was observed in the leaves of ‘NT212’ and ‘NT606’ under control conditions (Figure 1A_1_,A_2_). The sensitive cultivar ‘NT212’ exhibited peeling of the abaxial epidermis, loosely arranged parenchyma cells, and even cell lysis under H1, H2, H3, WH1, and WH2 treatments (Figure 1C_1_,D_1_,E_1_,F_1_,G_1_). Notably, the spatial damage in ‘NT212’ under WH2 was characterized by extensive abaxial epidermal peeling (PE), with disintegrated cells (DC) predominantly localized in the spongy parenchyma prior to palisade parenchyma collapse (Figure 1G_1_,H_1_). However, the tolerant cultivar ‘NT606’ only exhibited peeling of the abaxial epidermis under H2, H3, WH1, and WH2 (Figure 1D_2_,E_2_,F_2_,G_2_). In general, compared to ‘NT212’, ‘NT606’ demonstrated superior parenchyma organization under most different stress conditions (Figure 1).

**Figure 2:**
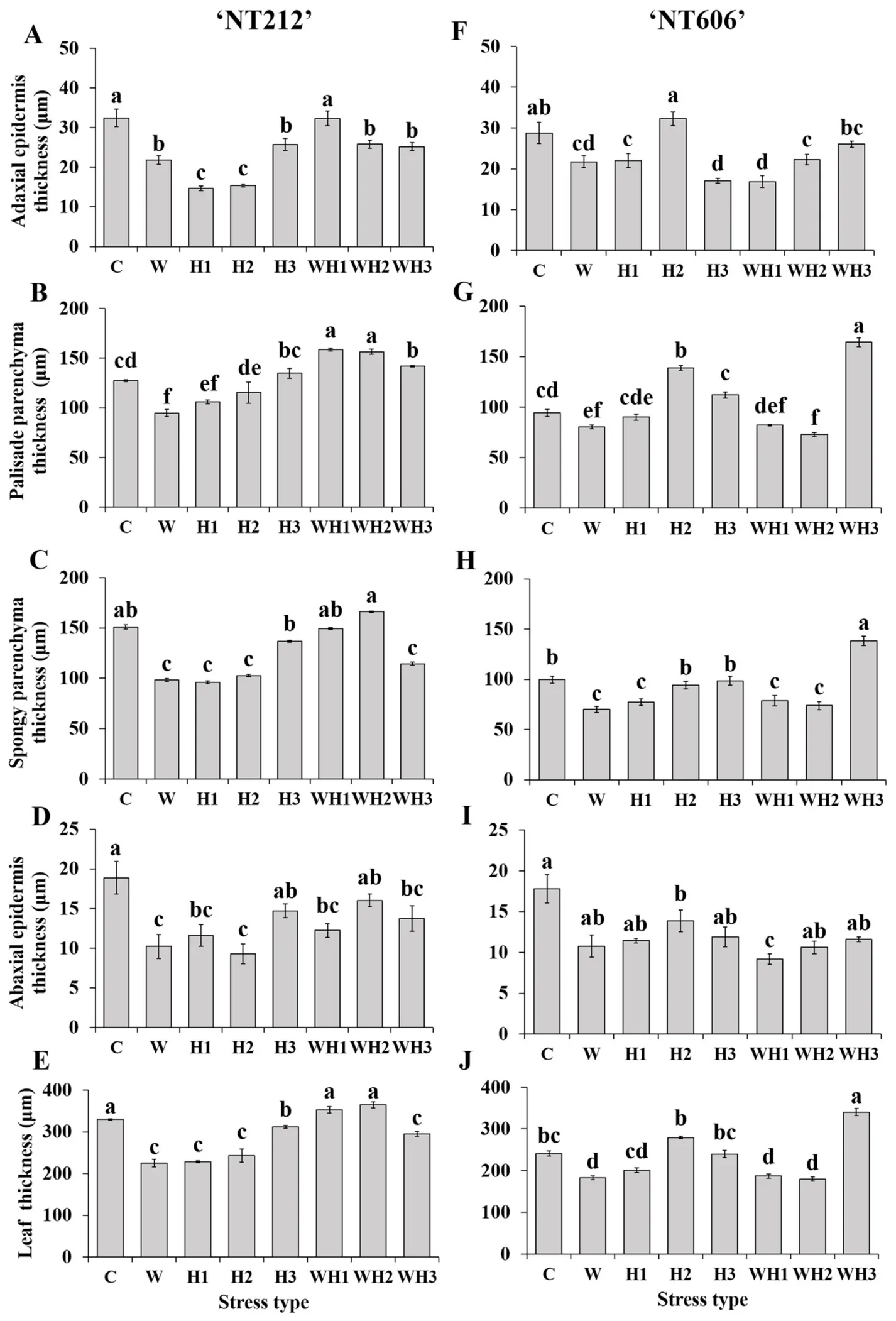
Adaxial epidermis thickness, palisade parenchyma thickness, spongy parenchyma thickness, abaxial epidermis thickness, and leaf thickness of (**A**–**E**) ‘NT212’ and (**F**–**J**) ‘NT606’ at control (C, 25/18 °C), waterlogging (W), heat stress (H1, 34/27 °C; H2, 38/31 °C; H3, 42/35 °C), and combined waterlogging and heat stress (WH1, waterlogging + 34/27 °C; WH2, waterlogging + 38/31 °C; WH3, waterlogging + 42/35 °C) for 48 h. Note: The results are mean ± SE (*n* = 3). Different lowercase letters above the bars indicate significant differences among different treatments (*p* < 0.05).

In ‘NT212’, the adaxial epidermis thickness was significantly reduced under all stress treatments except WH1 compared with control (Figure 2A). However, the thickness was significantly increased under WH1, WH2, and WH3 compared with H1 and H2 (Figure 2A). Palisade parenchyma thickness was significantly lower in ‘NT212’ under W and H1 than control, whereas all combined stress treatments (WH1, WH2, and WH3) caused a significant increased thickness as compared with control (Figure 2B). Furthermore, palisade parenchyma thickness was significantly greater under WH1, WH2, and WH3 than W, H1, and H2 (Figure 2B). Thickness of spongy parenchyma was significantly reduced in ‘NT212’ under W, H1, H2, and WH3 compared with control, which was significantly increased under H3, WH1, and WH2 compared to W, H1, and H2 (Figure 2C). The abaxial epidermis was significantly thinner in ‘NT212’ under W, H1, H2, WH1, and WH3 than control (Figure 2D). Notably, WH2 treatment resulted in a significant increase in abaxial thickness in ‘NT212’ compared to W and H2 (Figure 2D). Leaf thickness was significantly reduced in ‘NT212’ under the single stresses (W, H1, H2, and H3) compared to control (Figure 2E). Conversely, it was significantly increased in ‘NT212’ under WH1 and WH2 compared to the single stresses (W, H1, H2, and H3) (Figure 2E). In contrast, ‘NT606’ appeared to display a different adaptive strategy (Figure 2). Adaxial epidermis thickness was significantly reduced under W, H1, H3, WH1, and WH2 compared with the control (Figure 2F). Palisade parenchyma thickness was significantly higher in ‘NT606’ under H2 and WH3 than the control, which was significantly reduced under W and WH2 (Figure 2G). Palisade parenchyma thickness was significantly lower in ‘NT606’ under WH1 and WH2 but higher under WH3 compared with H2 and H3 (Figure 2G). For the spongy parenchyma, thickness was significantly decreased in ‘NT606’ under W, H1, WH1, and WH2, but increased under WH3 compared to the control (Figure 2H). Furthermore, the spongy parenchyma thickness was significantly reduced in ‘NT606’ under WH1 and WH2 but increased under WH3 compared with H2 and H3 (Figure 2H). Abaxial epidermis thickness was significantly reduced in ‘NT606’ only under WH1 compared with the control (Figure 2I). Leaf thickness was significantly lower in ‘NT606’ under W, WH1, and WH2, but significantly higher under WH3 compared to the control (Figure 2J). Additionally, leaf thickness was significantly decreased in ‘NT606’ under WH1 and WH2, but increased under WH3 as compared with H2 and H3 (Figure 2J).

### 3.2. Leaf Stomatal Characteristics

**Figure 3:**
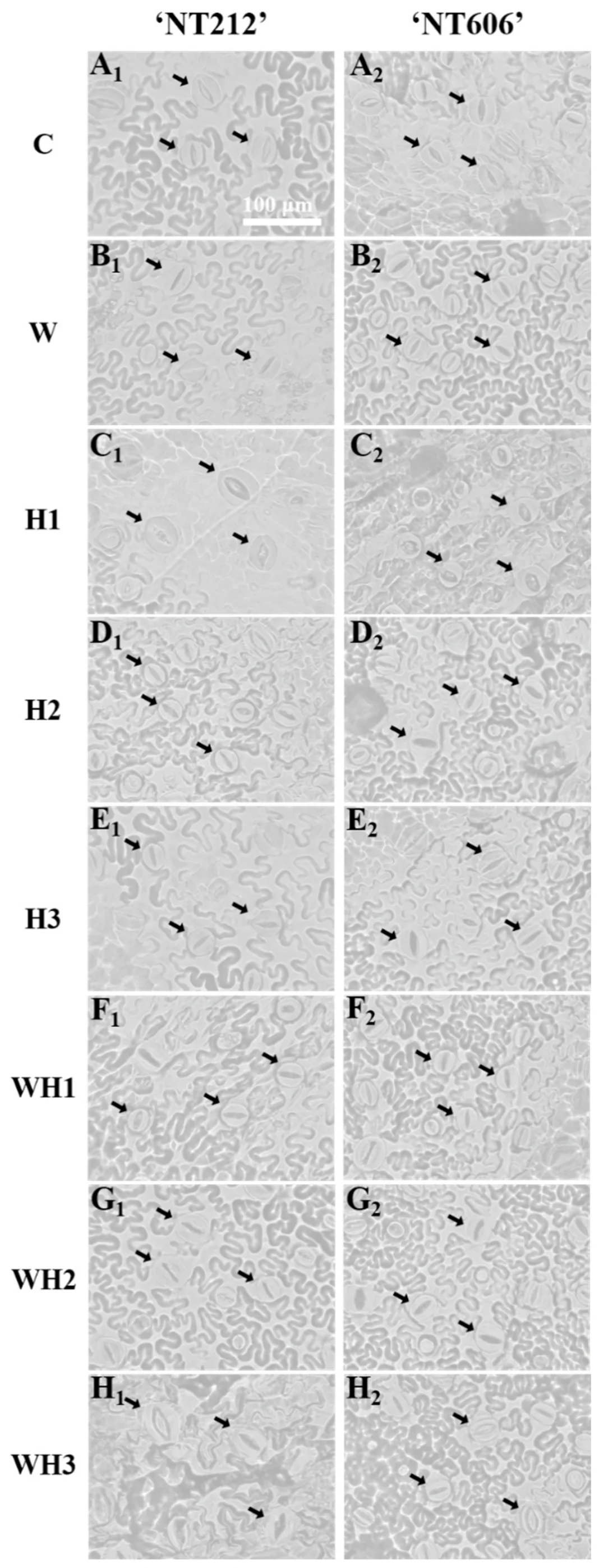
Stomatal morphology of ‘NT212’ and ‘NT606’ at (**A_1_**,**A_2_**) control (C, 25/18 °C), (**B_1_**,**B_2_**) waterlogging (W), (**C_1_**–**E_2_**) heat stress (H1, 34/27 °C; H2, 38/31 °C; H3, 42/35 °C), (**F_1_**–**H_2_**), and combined waterlogging and heat stress (WH1, waterlogging + 34/27 °C; WH2, waterlogging + 38/31 °C; WH3, waterlogging + 42/35 °C) for 48 h. The scale bar in (**A_1_**) represents 100 μm for all subfigures. Observations were made at 630 × magnification. Black arrows indicate the representative stomata used for quantitative analysis.

Stomatal analysis revealed distinct variations in stomatal morphology between ‘NT212’ and ‘NT606’ in response to waterlogging, heat stress, and combined stress (Figure 3). Both ‘NT212’ and ‘NT606’ displayed fully open stomata under control conditions (Figure 3A_1_,A_2_). By comparison, the W, H2, and H3 treatments resulted in varying degrees of stomatal closure in the two genotypes (Figure 3B_1_,B_2_,D_1_,D_2_,E_1_,E_2_). However, the H1 treatment promoted stomatal opening in both genotypes (Figure 3C_1_,C_2_). Furthermore, stomatal morphology exhibited a temperature-dependent shift under combined stress, where stomata appeared constricted under WH1, but evidenced opening under WH3, contrasting with the closure observed under W alone (Figure 3B_1_,B_2_,F_1_,F_2_,H_1_,H_2_).

**Figure 4:**
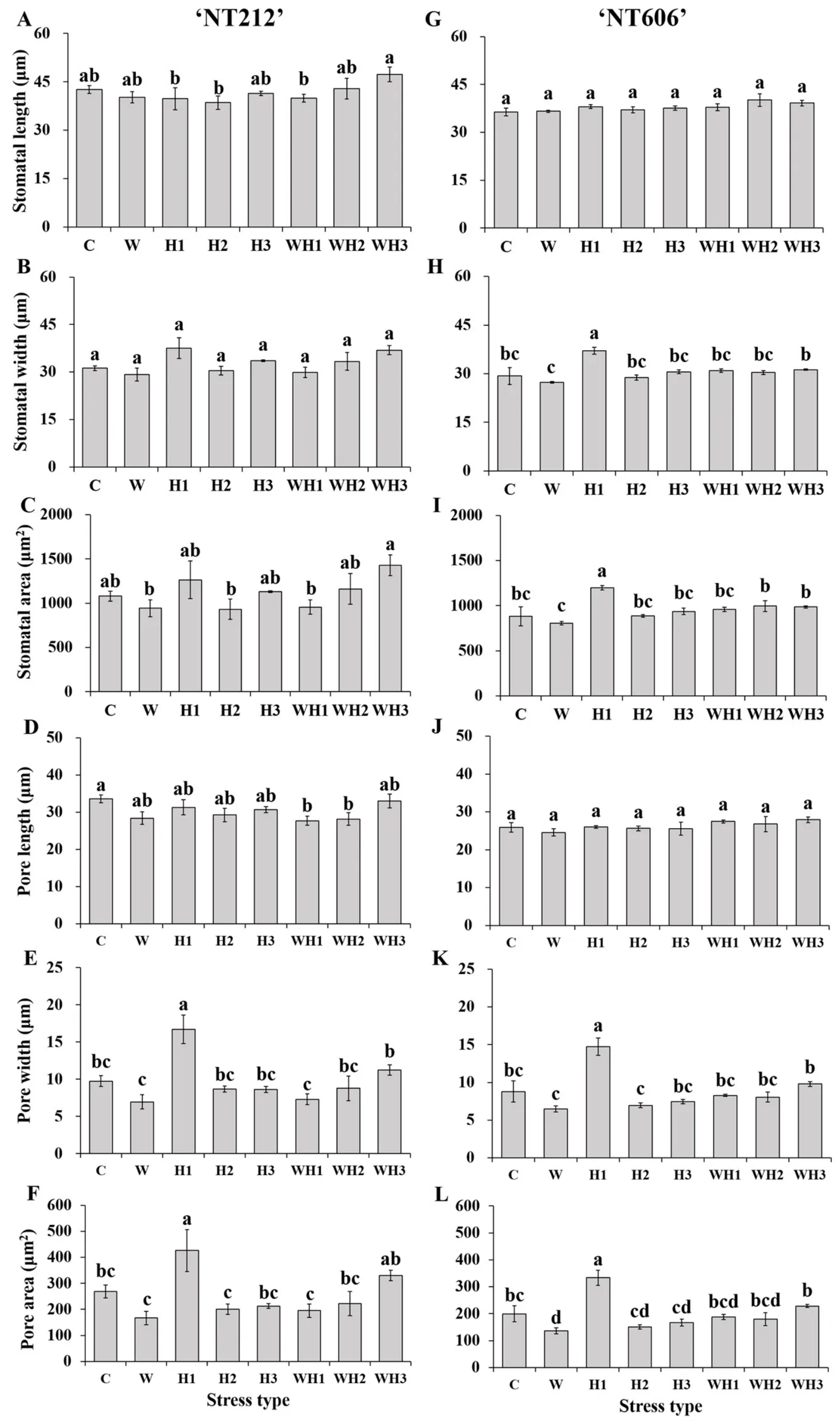
Stomatal length, width, and area and pore length, width, and area of (**A**–**F**) ‘NT212’ and (**G**–**L**) ‘NT606’ at control (C, 25/18 °C), waterlogging (W), heat stress (H1, 34/27 °C; H2, 38/31 °C; H3, 42/35 °C), and combined waterlogging and heat stress (WH1, waterlogging + 34/27 °C; WH2, waterlogging + 38/31 °C; WH3, waterlogging + 42/35 °C) for 48 h. Note: The results were mean ± SE (*n* = 27). Different lowercase letters above the bars indicate significant differences among different treatments (*p* < 0.05).

Pore width and area were significantly increased in ‘NT212’ under H1, even though stomatal length, width, area, and pore length showed no significant differences under any stress treatment compared to the control (Figure 4A–F). In ‘NT212’, pore width was significantly lower under WH1, WH2, and WH3, and pore area was significantly lower under WH1 and WH2 than H1 (Figure 4E,F). In ‘NT606’, stomatal width and area were significantly higher under H1 than control, even though stomatal and pore length under all stress treatments did not significantly differ from the control (Figure 4G–J). In ‘NT606’, while stomatal width was significantly higher under WH3 than W, it was significantly lower under WH1, WH2, and WH3 compared with H1 (Figure 4H). Similarly, stomatal area was higher in ‘NT606’ under WH2 and WH3 than W, which was significantly lower under WH1, WH2, and WH3 than H1 (Figure 4I). Pore width and area was significantly increased in ‘NT606’ under H1 as compared with control, which was significantly lower under WH1, WH2, and WH3 than H1 (Figure 4K,L).

### 3.3. Photosynthetic Pigment and Capacity

**Figure 5:**
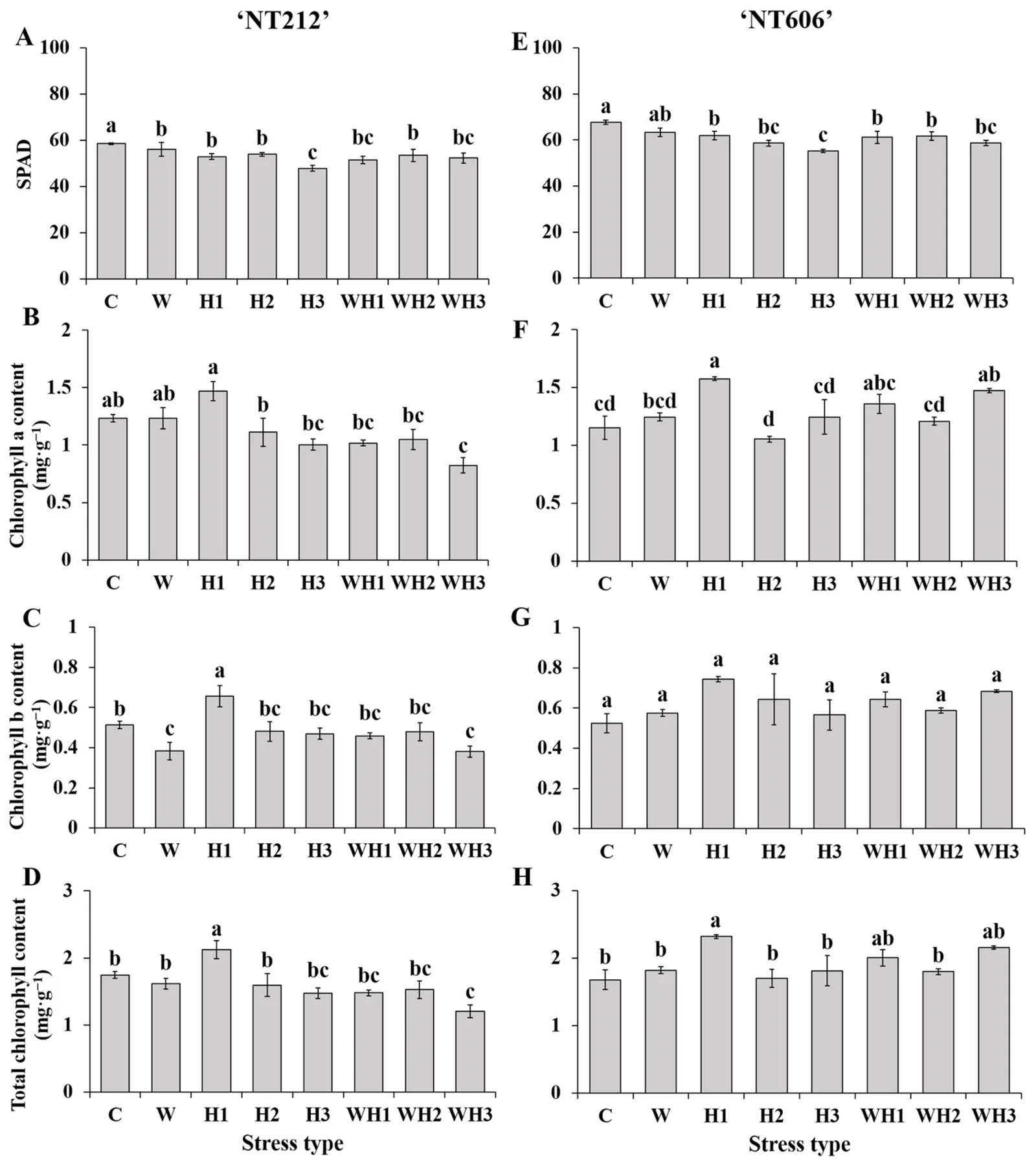
Leaf relative chlorophyll content, chlorophyll a content, chlorophyll b content, total chlorophyll content of (**A**–**D**) ‘NT212’ and (**E**–**H**) ‘NT606’ at control (C, 25/18 °C), waterlogging (W), heat stress (H1, 34/27 °C; H2, 38/31 °C; H3, 42/35 °C), and combined waterlogging and heat stress (WH1, waterlogging + 34/27 °C; WH2, waterlogging + 38/31 °C; WH3, waterlogging + 42/35 °C) for 48 h. Note: The results are mean ± SE (*n* = 3). Different lowercase letters above the bars indicate significant differences among different treatments (*p* < 0.05).

The leaf relative chlorophyll content was significantly reduced in ‘NT212’ under all stress treatments compared with control but significantly increased under WH2 compared with H3 (Figure 5A). Chlorophyll a content was significantly lower in ‘NT212’ under WH3 compared to control (Figure 5B). WH1, WH2, and WH3 significantly reduced chlorophyll a content compared with H1 in ‘NT212’ (Figure 5B). Chlorophyll b content was significantly increased under H1, but decreased in ‘NT212’ under W and WH3 compared with control (Figure 5C). In ‘NT212’, the total chlorophyll content was significantly increased under H1, but decreased under WH3 as compared with control (Figure 5D). The total chlorophyll content was significantly lower in ‘NT212’ under WH3 compared to W, H1 and H2 (Figure 5D).

Similarly, in ‘NT606’, the leaf relative chlorophyll content was significantly lower under all stress treatments compared to control, except for W, which was significantly higher under WH1 and WH2 than H3 (Figure 5E). For ‘NT606’, chlorophyll a content was significantly increased under H1 and WH3 compared with the control, which was higher under WH3 than H2 and H3 (Figure 5F). Total chlorophyll content was significantly increased in ‘NT606’ under H1 compared with the control, even though the chlorophyll b content showed no significant differences under all treatments (Figure 5G,H).

**Figure 6:**
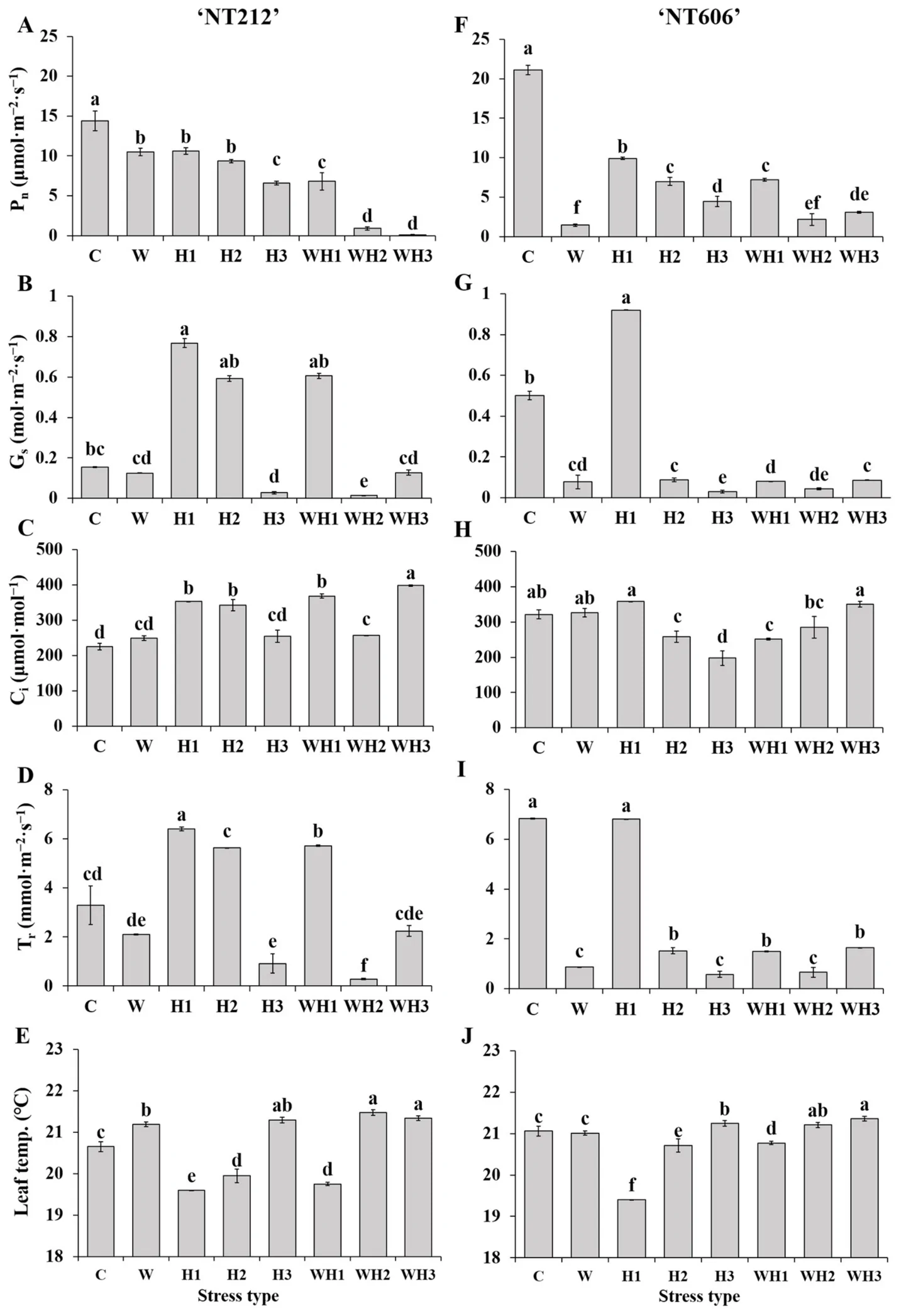
Net photosynthetic rate (P_n_), stomatal conductance (G_s_), intercellular CO_2_ concentration (C_i_), transpiration rate (T_r_), and leaf temperature of (**A**–**E**) ‘NT212’ and (**F**–**J**) ‘NT606’ at control (C, 25/18 °C), waterlogging (W), heat stress (H1, 34/27 °C; H2, 38/31 °C; H3, 42/35 °C), and combined waterlogging and heat stress (WH1, waterlogging + 34/27 °C; WH2, waterlogging + 38/31 °C; WH3, waterlogging + 42/35 °C) for 48 h. Note: The results are mean ± SE (*n* = 3). Different lowercase letters above the bars indicate significant differences among different treatments (*p* < 0.05).

‘NT212’ showed a significant decrease in net photosynthetic rate (P_n_) under multiple stress conditions, with a more pronounced reduction under combined stress as compared with the control (Figure 6A). In ‘NT212’, stomatal conductance (G_s_) was significantly higher under H1, but lower under H3 and WH2 than the control (Figure 6B). G_s_ was significantly lower in ‘NT212’ under WH2 and WH3 than H1 and H2 (Figure 6B). A significant increase in intercellular CO_2_ concentration (C_i_) was observed in ‘NT212’ under H1, H2, WH1, WH2, and WH3 as compared with the control (Figure 6C). For ‘NT212’, the C_i_ was significantly lower under WH2, but higher under WH3 compared with H1 and H2 (Figure 6C). A significant increase in transpiration rate (T_r_) was observed in ‘NT212’ under H1 and WH1 compared with the control, while it was significantly decreased under H3 and WH2 (Figure 6D). In ‘NT212’, leaf temperature was significantly higher under W, H3, WH2, and WH3, but lower under H1, H2, and WH1 than the control (Figure 6E). In ‘NT606, P_n_ was significantly reduced under the stress conditions compared to the control (Figure 6F). G_s_ was significantly higher in ‘NT606’ under H1, but lower under all other stress treatments than the control (Figure 6G). In ‘NT606’, C_i_ was significantly lower under H2, H3, and WH1 than the control, but higher under WH1, WH2, and WH3 than H3 (Figure 6H). Tᵣ was significantly reduced in ‘NT606’ under all stress treatments except the H1 compared with the control, and it was significantly lower under WH1, WH2, and WH3 than H1 (Figure 6I). In ‘NT606’, leaf temperature was significantly higher under H3, WH2, and WH3, but lower under H1, H2, and WH1 than the control (Figure 6J). A significant increase in leaf temperature was observed in ‘NT606’ under all combined stress treatments compared with H1 and H2 (Figure 6J).

### 3.4. Oxidative Stress

**Figure 7:**
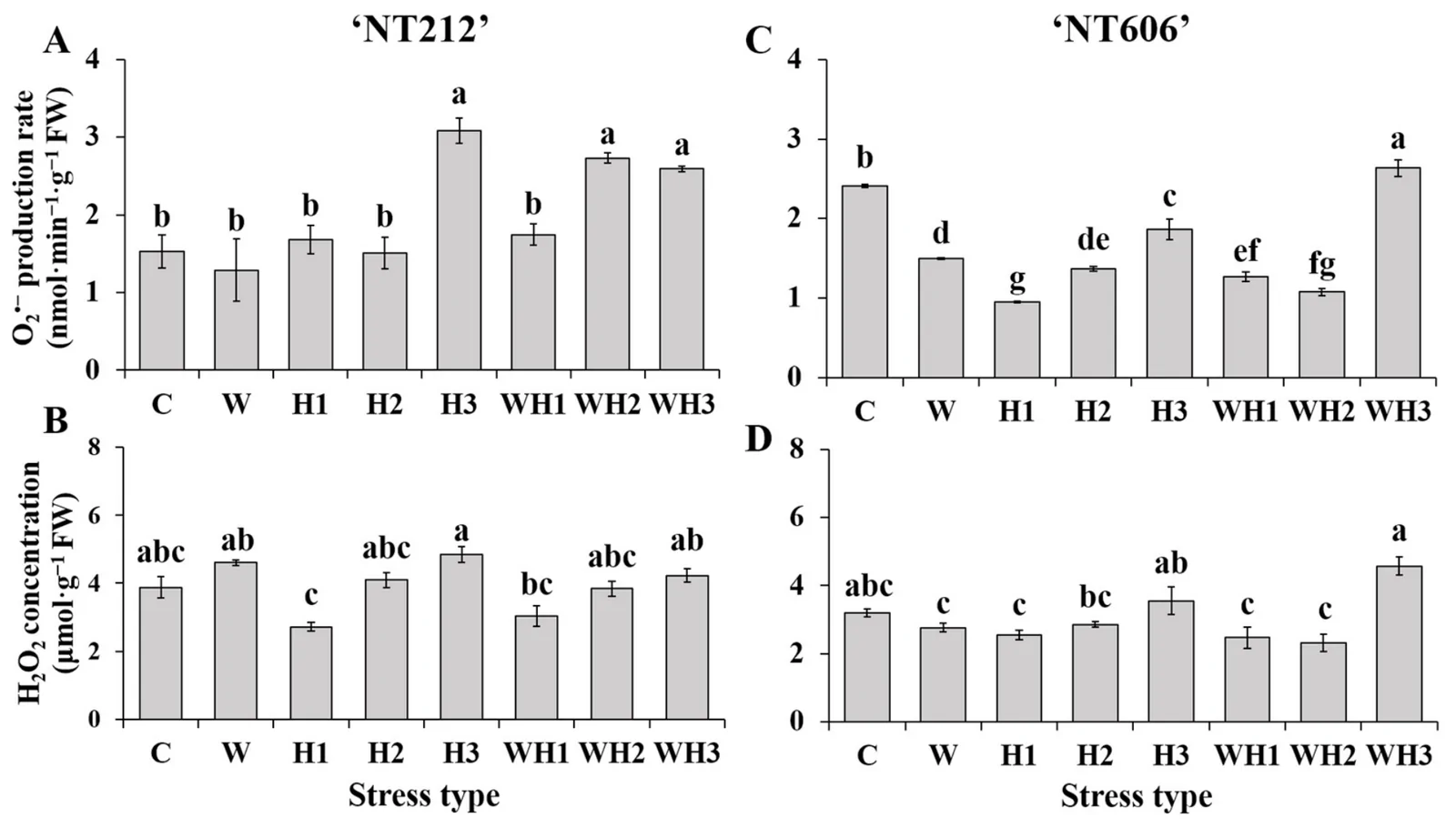
Leaf O_2_^•−^ production rate and H_2_O_2_ concentration of (**A**,**B**) ‘NT212’ and (**C**,**D**) ‘NT606’ at control (C, 25/18 °C), waterlogging (W), heat stress (H1, 34/27 °C; H2, 38/31 °C; H3, 42/35 °C), and combined waterlogging and heat stress (WH1, waterlogging + 34/27 °C; WH2, waterlogging + 38/31 °C; WH3, waterlogging + 42/35 °C) for 48 h. Note: The results are mean ± SE (*n* = 3). Different lowercase letters above the bars indicate significant differences among different treatments (*p* < 0.05).

Analysis of ROS metabolism revealed a significant alteration in ROS homeostasis (Figure 7). In ‘NT212’, O_2_^•−^ production rate was significantly increased under H3, WH2, and WH3 treatments compared to control, W, H1, and H2 (Figure 7A). Although H_2_O_2_ concentrations showed no significant differences in ‘NT212’ across all stress treatments compared with the control, it was significantly higher under H3 and WH3 compared to H1 (Figure 7B). By comparison, ‘NT606’ exhibited a relatively steady ROS accumulation (Figure 7C,D). A significant increase in O_2_^•−^ production rate was only observed in ‘NT606’ under WH3, while the other stress treatments showed a significant decrease compared to the control (Figure 7C). The O_2_^•−^ production rate was significantly decreased in ‘NT606’ under WH1 and WH2, but significantly increased under WH3 compared with H3 (Figure 7C). Moreover, H_2_O_2_ concentration was higher in ‘NT606’ under only WH3 than W, H1, and H2, even though it did not show significant differences under all the stress treatments compared with the control (Figure 7D).

### 3.5. Stress-Responsive Transcript Expression

**Figure 8:**
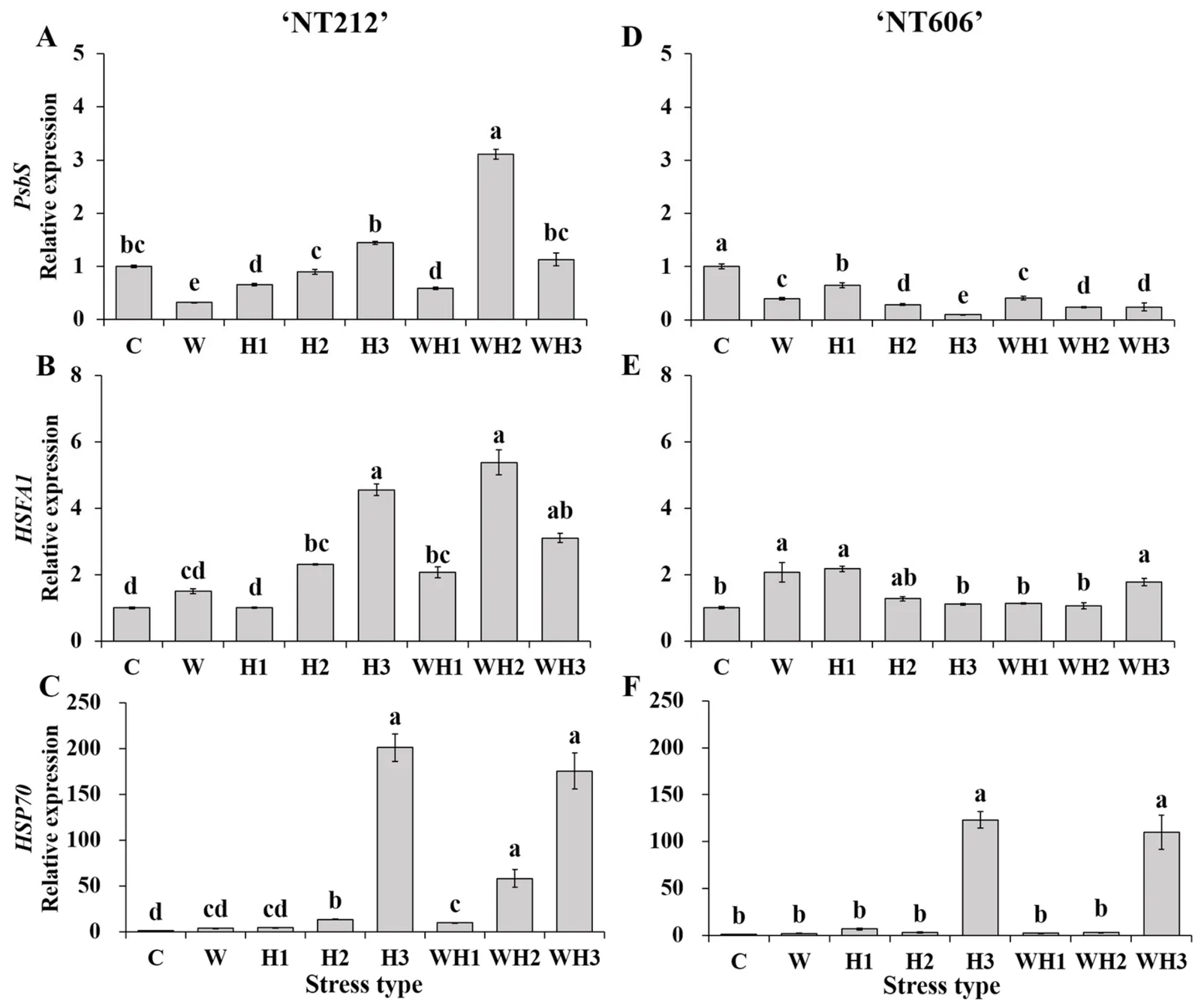
Relative expression levels of *PsbS* (Photosystem II subunit S), *HSFA1* (heat shock transcription factor A1), and *HSP70* (heat shock protein 70) in (**A**–**C**) ‘NT212’ and (**D**–**F**) ‘NT606’ at control (C, 25/18 °C), waterlogging (W), heat stress (H1, 34/27 °C; H2, 38/31 °C; H3, 42/35 °C), and combined waterlogging and heat stress (WH1, waterlogging + 34/27 °C; WH2, waterlogging + 38/31 °C; WH3, waterlogging + 42/35 °C) for 48 h. Note: The data are mean ± SE (*n* = 3). Different lowercase letters above the bars indicate significant differences among different treatments (*p* < 0.05).

Transcript expression profiles markedly varied under stress between the two genotypes (Figure 8). In ‘NT212’, the expression of *PsbS* was significantly upregulated only under WH2, which was downregulated under W, H1, and WH1 compared to the control (Figure 8A). Furthermore, *PsbS* expression was significantly higher in ‘NT212’ under WH2 than all single stress treatments (Figure 8A). The expression of *HSFA1* was significantly higher in ‘NT212’ under H2, H3, WH1, WH2, and WH3 than the control, which was significantly higher under WH1, WH2, and WH3 than H1 (Figure 8B). The expression of *HSP70* was strongly induced in ‘NT212’ under H2, H3, WH1, WH2, and WH3, with particularly high levels of H3, WH2, and WH3 (Figure 8C). In ‘NT606’, *PsbS* expression was significantly downregulated under all stress treatments compared to control, which was significantly lower under WH1, WH2, and WH3 than H1 (Figure 8D). *HSFA1* expression was significantly higher in ‘NT606’ under W, H1, and WH3 than the control, which was lower under WH1 and WH2 than W and H1 (Figure 8E). *HSP70* expression was significantly upregulated in ‘NT606’ under H3 and WH3 as compared with the other treatments (Figure 8F).

### 3.6. Plant Growth Under Conditions of Waterlogging and Heat Stress Combination

‘NT212’ showed a significant decline in plant height under all stress treatments except for H1 compared to the control, which was most pronounced under WH2 and WH3 (Figure S1A). Plant fresh weight was significantly lower in ‘NT212’ under WH2 than the control, even though plant dry weight showed no significant differences under all treatments (Figure S1B,C). In contrast, ‘NT606’ demonstrated strong tolerance, maintaining plant heights comparable to control across all single stress and combined stress conditions, except for WH3 (Figure S1D). Dry weight of ‘NT606’ was significantly lower under H2, WH1, and WH2 than H1, although no significant difference was observed in fresh weight (Figure S1E,F). Distinct phenotypic alterations were also observed between the two cultivars under stress conditions (Figure S1G). ‘NT212’ exhibited severe wilting under the H2, H3, WH1, WH2, and WH3 conditions, while ‘NT606’ demonstrated less phenotypic inhibition, with severe wilting being observed primarily under H3 and WH3 (Figure S1G).

### 3.7. Effects of Individual Factors and Their Interactions on the Parameters and Hierarchical Clustering Analysis of Treatments

**Figure 9:**
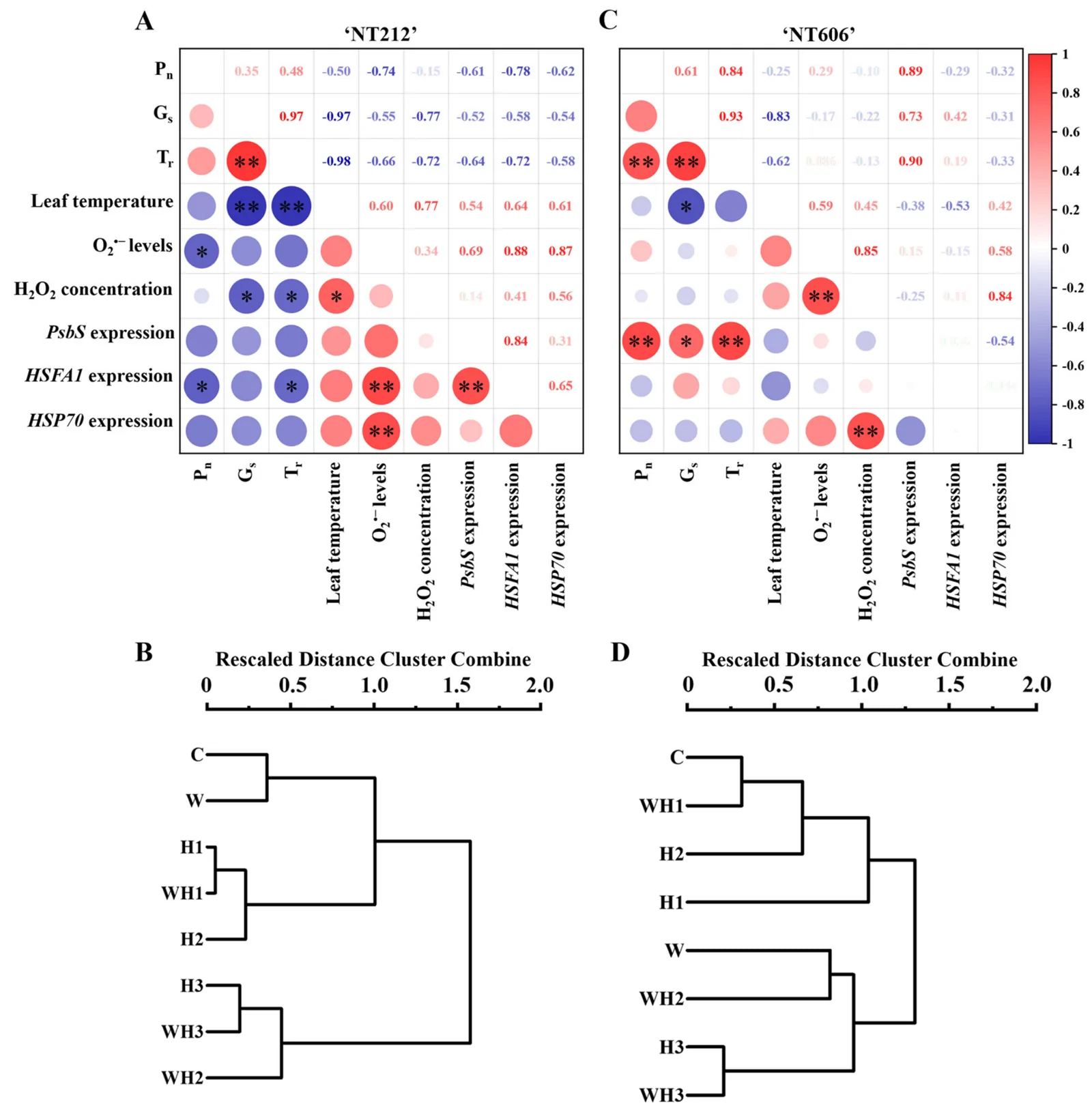
Correlation analysis of the measured parameters and hierarchical clustering analysis based on squared Euclidian distance on a treatment × parameter matrix in (**A**,**B**) ‘NT212’ and (**C**,**D**) ‘NT606’ at control (C, 25/18 °C), waterlogging (W), heat stress (H1, 34/27 °C; H2, 38/31 °C; H3, 42/35 °C), and combined waterlogging and heat stress (WH1, waterlogging + 34/27 °C; WH2, waterlogging + 38/31 °C; WH3, waterlogging + 42/35 °C). Note: The key 9 parameters were shown in the x and y axis. The numbers in the square represent the correlation coefficient. * and ** represent *p* < 0.05 and *p* < 0.01, respectively. Abbreviations: P_n_, net photosynthetic; G_s_, stomatal conductance; T_r_, transpiration rate; *PsbS*, Photosystem II subunit S; *HSFA1*, heat shock transcription factor A1; *HSP70*, heat shock protein 70.

In ‘NT212’, the P_n_ showed a significantly negative correlation with O_2_^•−^ production rate (Figure 9A). The G_s_ and T_r_ in ‘NT212’ were found to be significantly negatively correlated with leaf temperature and H_2_O_2_ concentration (Figure 9A). For ‘NT212’, the O_2_^•−^ production rate was significantly positively correlated with *HSFA1* and *HSP70* expression (Figure 9A). In contrast, the P_n_, G_s_, and T_r_ in ‘NT606’ showed significantly positive correlations with *PsbS* expression (Figure 9C). For ‘NT606’, G_s_ was found to be significantly negatively correlated with leaf temperature (Figure 9C). H_2_O_2_ concentration in ‘NT606’was observed to be significantly positively correlated with O_2_^•−^ production rate and *HSP70* expression (Figure 9C). Hierarchical clustering based on all the measured indices showed that the two genotypes were differently affected by the climatic factors (Figure 9B,D). For ‘NT212’, the treatments were clustered into three groups when the rescaled distance was 1.0 (Figure 9B). The first main group consisted of the plants under the control and W (Figure 9B). The second group contained the plants under H1, H2, and WH1, while the third group comprised the plants under H3, WH2, and WH3 (Figure 9B). In contrast, for ‘NT606’, the treatments formed three main groups at the same rescaled distance (Figure 9D). The first main group consisted of the plants under the control, H2, and WH1 (Figure 9D). The second group contained only the plants under H1, while the third group comprised the plants under W, H3, WH2, and WH3 (Figure 9D).

## 4. Discussion

### 4.1. Combined Waterlogging and Heat Stress Inflicts More Severe Synergistic Damage than Individual Stress with Genotypic Difference

When plants face combined environmental constraints, the interaction often triggers unique, non-additive physiological responses where the compound effect significantly exceeds the sum of individual stresses [3,6]. Leaf anatomy forms the structural foundation for photosynthesis, governing both light interception and CO_2_ diffusion to carboxylation sites [21]. While ‘NT212’ exhibited no significant damage to leaf anatomy under waterlogging stress alone, this sensitive cultivar generally showed slight abaxial epidermis peeling under heat stress (H1, H2, and H3), with cell lysis observed only under H3 (Figure 1C_1_,D_1_,E_1_). Moreover, the anatomical breakdown in ‘NT212’ was severe when exposed to combined stress, which is mainly attributed to parenchyma degeneration induced by spongy parenchyma cell lysis (Figure 1F_1_,G_1_). This selective degradation of the spongy parenchyma suggests a loss of the internal gas exchange area, creating a severe physical barrier to CO_2_ diffusion [40,41,42]. This structural vulnerability in ‘NT212’ under combined stress directly translated to a precipitous decline in P_n_ under WH2 and WH3 compared with WH1 (Figure 6A). The selective degradation of the spongy parenchyma leads to the collapse of intercellular airspaces, which are normally filled with gas and serve as low resistance pathways for CO_2_ diffusion [43]. Upon cell lysis, these airspaces become flooded with cellular contents, converting the rapid gas-phase diffusion into an extremely slow liquid-phase diffusion [21,42]. Consequently, a severe physical barrier to CO_2_ transport is created, dramatically reducing parenchyma conductance and limiting CO_2_ availability for photosynthesis [40,41,42]. Furthermore, plants faced a severe physiological conflict between retaining water and transpiration cooling, forcing stomata into extreme states that ultimately fail to maintain gas exchange and carbon assimilation under combined stress [44]. In contrast, ‘NT606’ exhibited superior ability to alleviate this synergistic damage. While ‘NT212’ suffered cellular lysis, ‘NT606’ consistently maintained a superior level of parenchyma organization under combined stress (WH2), with structural alterations largely confined to mild abaxial epidermis peeling (Figure 1G_2_). Moreover, ‘NT606’ maintained plant height better across all single stress and most combined stress (WH1 and WH2) conditions, whereas ‘NT212’ suffered severe growth inhibition under combined conditions (Figure S1A,D). The aggravated growth decline under combined stress was mechanistically linked to a more severe leaf anatomy damage compared to single-stress conditions (Figure 1, Figure 2, Figure 5 and Figure 6).

Abiotic stresses invariably lead to the overproduction of ROS [45], but the combination of waterlogging and heat appeared to create a highly toxic cellular environment. Under heat stress, ROS burst damages chloroplasts and mitochondria, disrupting photosynthetic and respiratory functions; this damage is further amplified by inter-organellar crosstalk, ultimately leading to programmed cell death and whole-plant growth suppression [46]. In ‘NT212’, the O_2_^•−^ production rate under combined stress (WH2 and WH3) was significantly higher than under individual stress (W, H1, and H2) (Figure 7A), indicating that the simultaneous disruption of the photosynthetic electron transport chain and mitochondrial respiration may overwhelm the plant’s basal antioxidant capacity [47]. The expression of *HSP70* was strongly induced in ‘NT212’, particularly under high levels of H3, WH2, and WH3 compared with the control (Figure 8C). Consequently, this excessive ROS accumulation necessitates a stronger molecular defense response, evident in the massive upregulation of the major molecular chaperone HSP70 to combat misfolded proteins [48]. In accordance, the O_2_^•−^ production rate was positively correlated with the expression of *HSP70* expression in ‘NT212’ (Figure 9A), reflecting a state of severe oxidative distress. The uncontrolled ROS burst overwhelms basal cellular defenses, triggering insufficient hyper-activation of HSP70 chaperones that fails to prevent irreversible protein denaturation and cellular collapse [46,48]. In contrast, a significant decrease in O_2_^•−^ production rate was observed in ‘NT606’ under all stress treatments except for WH3, compared with control (Figure 7C), and *HSP70* expression was significantly upregulated in ‘NT606’ under H3 and WH3 as compared with the other treatments (Figure 8F). This indicated that ‘NT606’ could prevent severe oxidative damage by efficiently detoxifying ROS. Overall, our results demonstrate that combined waterlogging and heat stress trigger a synergistic damage cascade in tomato plants that is fundamentally more detrimental than either stress applied individually, ultimately translating into profound growth inhibition.

### 4.2. Escalating Heat Intensity Under Combined Stress Exacerbates the Damage Cascade from Physiological to Molecular Levels

Although combined stress is inherently more detrimental than single stress, the extent of this damage is highly dependent on stress intensity [49]. Here, escalating temperature during waterlogging (from WH1 to WH3) progressively exacerbated damage across morphological, physiological, and molecular levels, underscoring a critical shift in plant fate—from active defense to complete dysfunction. For instance, in ‘NT212’, WH1 clustered with the H1 and H2, indicating a manageable level of impairment. However, as the intensity increased to WH2 and WH3, these treatments were reclassified into a distinct cluster characterized by severe physiological collapse (Figure 9B). This progressive shift in clustering visually demonstrates that escalating stress intensity progressively deepens the systemic damage, eventually overwhelming plant defenses [49].

From an anatomical and physiological perspective, the escalating stress intensity drove profound shifts in leaf structure and stomatal regulation. In ‘NT212’, as the stress intensified from WH1 to WH2, anatomical breakdown worsened, manifesting as peeled epidermis, loosely arranged parenchyma, and eventually complete cell lysis (Figure 1F_1_,G_1_). As the combined stress intensified, these types of parenchyma could no longer withstand the compound pressure, ultimately undergoing structural collapse that resulted in a significant reduction in ‘NT212’ under WH3 in both palisade parenchyma and spongy parenchyma compared with WH1 and WH2 (Figure 2B,C). Specifically, extensive abaxial epidermal peeling compromised the leaf’s hydraulic integrity [40,50]. Moreover, degeneration of the spongy parenchyma and poor differentiation between palisade and spongy parenchyma further imposed a severe physical barrier to CO_2_ diffusion [40,41]. Conversely, ‘NT606’ displayed a highly regulated physical response to this escalating heat stress gradient. Under WH1 and WH2, ‘NT606’ actively avoided this severe cell lysis (Figure 1F_2_,G_2_). The ‘NT606’ exhibited a significant increase in palisade parenchyma thickness, spongy parenchyma thickness, and total leaf thickness only under high combined stress intensity (WH3) compared to the control (Figure 2G,H,J). Similar increases in palisade and spongy parenchyma thickness have been reported for *Chrysanthemum zawadskii* under waterlogging stress for 10 days [51] and for soybean under heat stress at 38/28 °C for 14 days [52] compared with the respective controls. These suggested high combined waterlogging and heat stress intensity could drive leaf anatomical thickening to protect the plants from stress conditions.

Stomatal conductance across the stress gradient illustrates the escalating damage to plants. As outlined above, under combined stress, the plant faces a physiological dilemma: close stomata to conserve water (a signal from the waterlogged roots) or open them to cool the leaves to respond to heat [7,44]. In ‘NT212’, this may lead to the observed significant drop in G_s_ under WH2 and WH3 compared to WH1, which showed no difference from the control (Figure 6B). On one hand, this decline is attributed to the substantial increase in VPD under high temperatures, which forces stomatal closure to prevent severe water loss [24]. On the other hand, in the waterlogged environment, the anoxic conditions severely damage the root system, drastically reducing its water absorption capacity and placing the shoot in extreme physiological drought [8]. Correlation analysis revealed a significant negative correlation between G_s_ and leaf temperature in both genotypes (Figure 9). The impaired roots could not supply water fast enough and the elevated leaf temperature was caused by decreased G_s_, which led to severe and irreversible wilting in ‘NT212’ (Figure S1G). Interestingly, the G_s_ either showed no significant differences in ‘NT212’ under all combined stress compared to the control, whereas C_i_ was significantly increased or maintained at a higher level (Figure 6C). A high C_i_ coupled with a low P_n_ is a classic indicator of non-stomatal limitation [53]. This demonstrates that the photosynthetic machinery itself, i.e., the enzymes of the Calvin cycle (like Rubisco) and the electron transport chain, was severely damaged and unable to fix the CO_2_ that was available within the leaf [54,55]. In contrast, while ‘NT606’ also exhibited stomatal limitations, it maintained a relatively stable or even decreased C_i_ under all combined stress conditions compared to the control (Figure 6H). This suggests that its biochemical capacity was much better preserved, aligning directly with its superior anatomical integrity.

**Figure 10:**
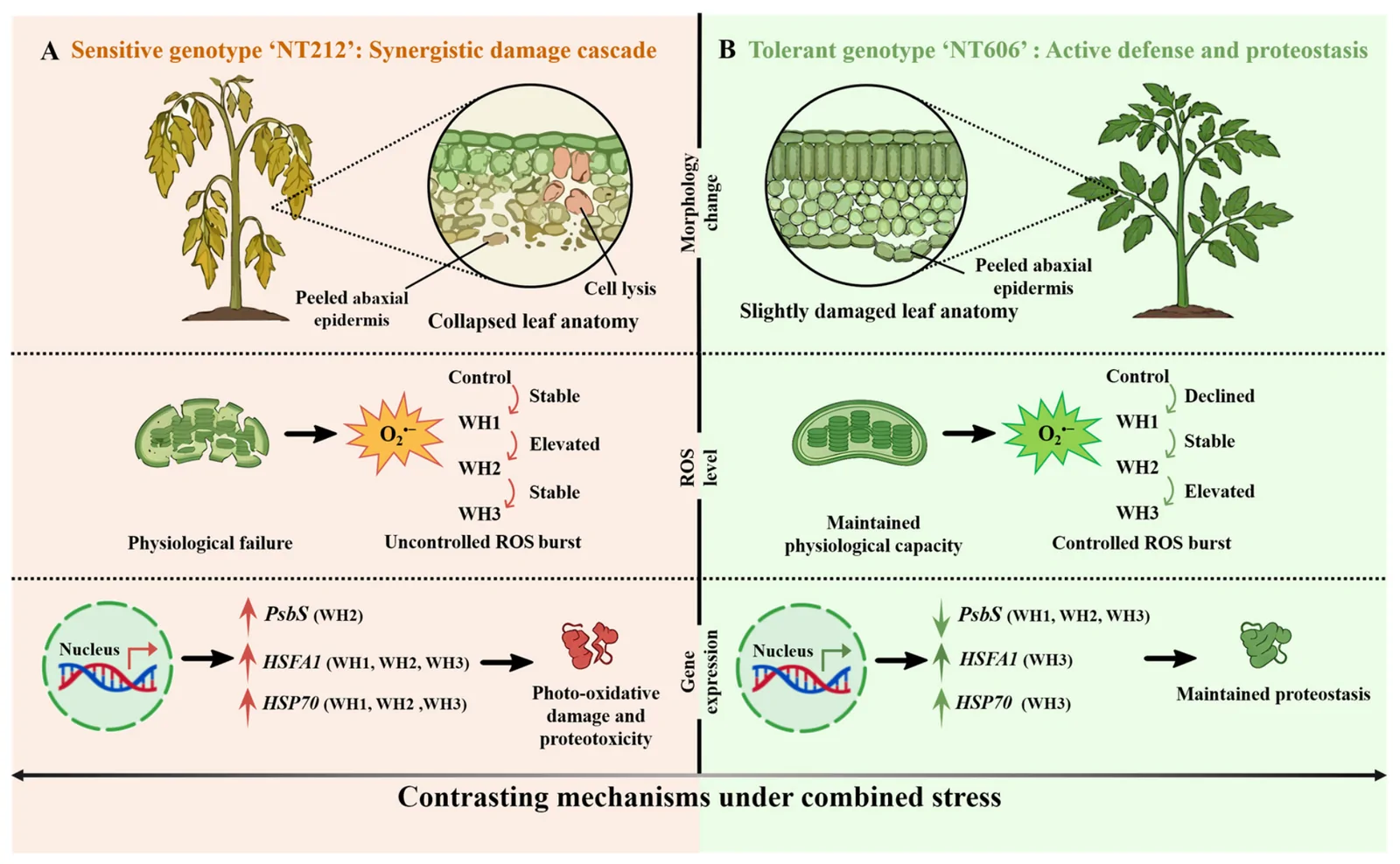
Potentially regulatory mechanism in (**A**) ‘NT212’ and (**B**) ‘NT606’ responding to combined waterlogging and heat stress (WH1, waterlogging + 34/27 °C; WH2, waterlogging + 38/31 °C; WH3, waterlogging + 42/35 °C). Abbreviations: P_n_, net photosynthetic rate; G_s_, stomatal conductance; C_i_, intercellular CO_2_ concentration; *PsbS*, Photosystem II subunit S; *HSFA1,* heat shock transcription factor A1; *HSP70*, heat shock protein 70.

The progression from WH1 to WH3 triggered an uncontrolled oxidative burst in ‘NT212’ that appeared to overwhelm its defense network (Figure 7 and Figure 8). The O_2_^•−^ production rate was significantly increased under WH2 and WH3 compared with WH1, which showed no significant differences with the control (Figure 7A). The ROS burst could damage chloroplasts and mitochondria under heat stress [46]. Alongside this oxidative distress, a significant molecular distinction between the two genotypes was the divergent regulation of *PsbS* (Figure 8A,D). *PsbS* is a core component of non-photochemical quenching (NPQ) that dissipates excess light energy [33]. As the stress intensified to WH2 and WH3, the sensitive ‘NT212’ significantly upregulated *PsbS* expression compared with WH1 (Figure 8A). However, sustained or over-activated NPQ can exacerbate energetic deficits and restrict photosynthetic efficiency [56]. Furthermore, the continuous translation of such abundant proteins under severe stress imposes a massive energetic burden and overwhelms the compromised cellular folding machinery [15]. This inevitably leads to the accumulation of misfolded or unassembled proteins, triggering severe proteotoxicity that fatally disrupts cellular homeostasis [15,48]. Conversely, *PsbS* expression in ‘NT606’ was significantly downregulated under WH2 and WH3 compared with WH1 (Figure 8D). ‘NT606’ likely displayed an active metabolic conservation strategy by downregulating *PsbS* expression as the stress intensified from WH1 to WH2 and WH3. By limiting NPQ-mediated energy loss, this preserved limited photochemical capacity [57]. Moreover, the expression of *HSP70* was significantly upregulated in ‘NT212’ under WH2 and WH3 compared to WH1, surging to exceptionally high levels under WH3 (Figure 8C), potentially reflecting a response to mitigate protein denaturation driven by extreme heat and ROS [48]. In contrast to ‘NT212’, ‘NT606’ only exhibited a significant increase in O_2_^•−^ under WH3 compared with WH1 and WH2 (Figure 7C), and correspondingly mounted a highly modulated *HSP70* response precisely at this stage (Figure 8F). This response could suggest that ‘NT606’ deploys its heat shock response to maintain proteostasis [15]. Ultimately, these multi-level molecular and physiological failures in ‘NT212’ culminated in compromised growth—manifesting as dramatic reductions in fresh weight under WH2 compared with the control and widespread, severe wilting under WH2 and WH3 (Figure S1B,G). Conversely, ‘NT606’ demonstrated robust phenotypic resilience, with severe wilting and height reduction delayed until WH3 (Figure S1D,G). Generally, the expression of *PsbS* was upregulated under WH2, while the expression of *HSFA1* and *HSP70* was upregulated in the sensitive genotype under the three combined stressors compared to the control (Figure 10A). However, the magnitude of this defense response was insufficient to cope with the medium and high intensities of stress (WH2 and WH3). This led to the irreversible denaturation of numerous functional proteins (including PsbS, HSFA1, and HSP70) and the formation of toxic aggregates, which in turn induced a massive accumulation of O_2_^•−^ and chloroplast damage (Figure 10A). Ultimately, severe morphological changes due to oxidative damage and anatomical defects in the sensitive genotype were observed under WH2 and WH3 (Figure 10A). In contrast, the tolerant genotype exhibited greater physiological resilience, maintaining better leaf parenchyma integrity and ROS homeostasis (Figure 10B). Notably, *HSFA1* and *HSP70* were strongly induced in the tolerant genotype only under the high stress intensity (WH3) compared with the control, coinciding with a substantial accumulation of O_2_^•−^ specifically (Figure 10B). This strategy of activating core defenses only at critical thresholds reveals a sophisticated molecular adaptation mechanism in the tolerant genotype under combined stress.

## 5. Conclusions

The coordination of ROS homeostasis and stress-responsive gene expression is fundamental to how tomato plants cope with concurrent waterlogging and heat waves. Compared to individual stress, combined stress triggers an amplified oxidative burst and synergistic degradation of leaf parenchyma and photosynthetic capacity, which escalates as temperatures intensify. However, the tolerant genotype successfully defends against these extremes by preserving structural integrity and restricting ROS overproduction, thereby avoiding the irreversible spongy parenchyma cell lysis seen in the sensitive cultivar. Molecularly, this resilience is orchestrated by the precise modulation of *PsbS*, *HSFA1*, and *HSP70*, which collectively balance photoprotective energy dissipation and cellular protein stability. These mechanisms, integrating traits of sensitive and tolerant tomato genotypes under combined waterlogging and heat stress, provide a vital framework for breeding climate-resilient crops.

## Data Availability

Data are contained within the article or Supplementary Material.

## Supplementary Materials

The following supporting information can be downloaded at https://www.mdpi.com/article/10.3390/antiox15091210/s1: Figure S1: Plant height, fresh weight and dry weight of (A–C) ‘NT212’ and (D–F) ‘NT606’ and (G) morphological changes at control (C, 25/18 °C), waterlogging (W), heat stress (H1, 34/27 °C; H2, 38/31 °C; H3, 42/35 °C), combined waterlogging and heat stress (WH1, waterlogging + 34/27 °C; WH2, waterlogging + 38/31 °C; WH3, waterlogging + 42/35 °C) for 48 h. Table S1: qRT-PCR primer sequence information.
