# Supplementary material for: Enhanced Levels of Combined Waterlogging and Heat Stress Exacerbate Anatomical Damage, Photosynthetic Impairment, and Oxidative Stress in Tomato
Source: Antioxidants (Basel). 2026 Sep 20;15(9):1210. doi: 10.3390/antiox15091210 (PMC13603622; doi:10.3390/antiox15091210)
Supplement: Supplementary file 1 [file antioxidants-15-01210-s001.zip › antioxidants-4445885-supplementary.pdf]

Table S1. Sequences of qRT-PCR primers

Abbreviations of gene names	Full names of genes	Gene ID (ITAG4.0)	Sequences of forward primers (5'-3')	Sequences of reverse primers (5'-3')
PsbS	*Photosystem II subunit S*	Solyc06g060340	ACTCAGTGAAGGAGGTCCATTG	TTGTGCCAATGCTCCTTTCC
HSFA1	*heat shock transcription factor A1*	Solyc08g005170	CTTCAGGTCGTGTTTCGGGA	TGCATCTCAGAAGTGGCTGG
HSP70	*heat shock protein 70*	Solyc11g020040	CGGTGGCAGTAATGGAAGGT	CACCACCGATTGCCTTTTGG
Actin (reference)	/	Solyc03g078400	CTCTACATACTTGAGAGGTGCC	AGACGAGGAGAAAACATCACAA
