# Supplementary material for: Enhanced Levels of Combined Waterlogging and Heat Stress Exacerbate Anatomical Damage, Photosynthetic Impairment, and Oxidative Stress in Tomato
Source: Antioxidants (Basel). 2026 Sep 20;15(9):1210. doi: 10.3390/antiox15091210 (PMC13603622; doi:10.3390/antiox15091210)
Supplement: Supplementary file 1 [file antioxidants-15-01210-s001.zip › Supplementary figure-rong.pdf]

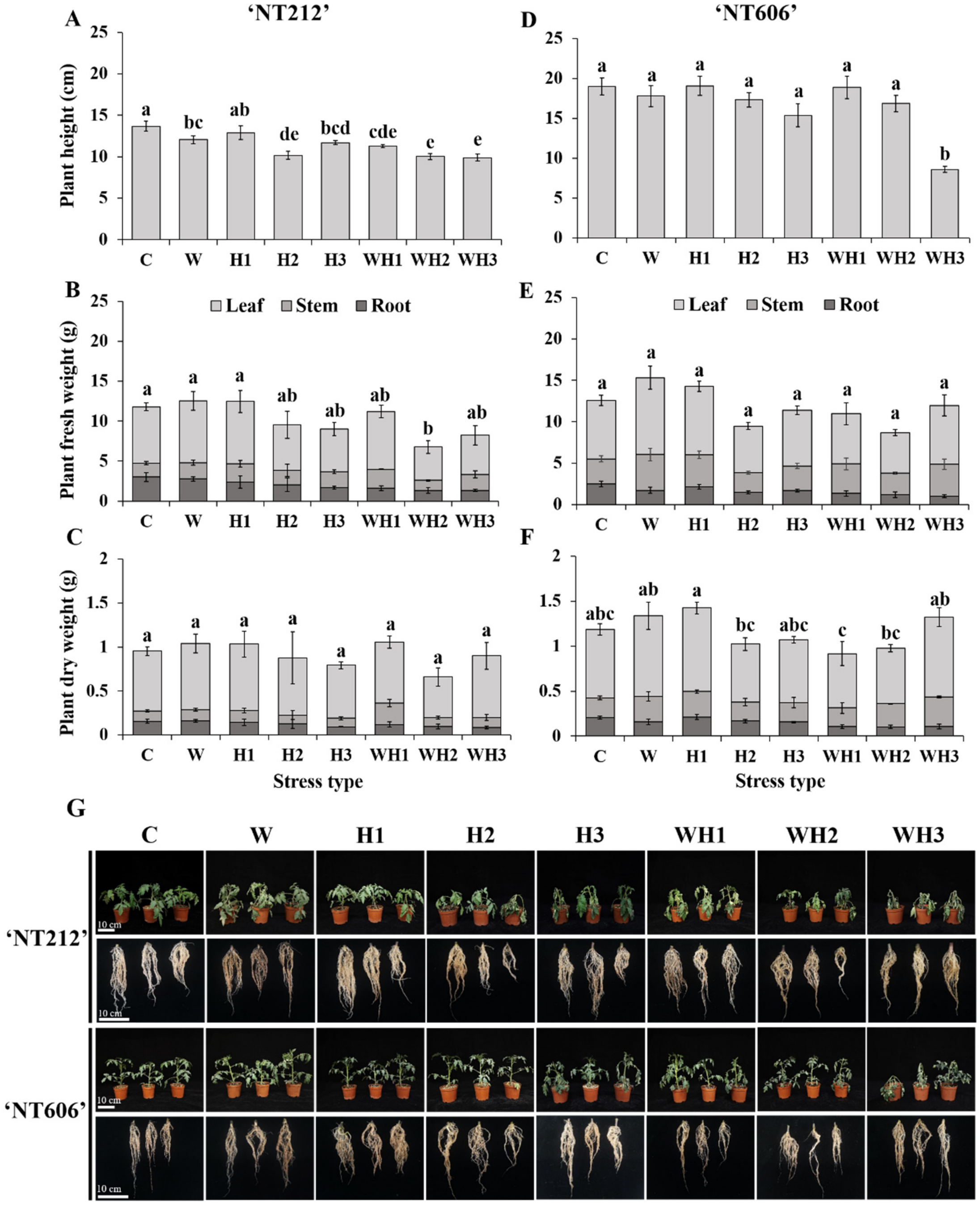

Figure S1. Plant height, fresh weight and dry weight of **(A, B, C)** 'NT212' and **(D, E, F)** 'NT606' and **(G)** morphological changes at control (C, 25/18°C), waterlogging (W), heat stress (H1, 34/27°C; H2, 38/31°C; H3, 42/35°C), combined waterlogging and heat stress (WH1, waterlogging + 34/27°C; WH2, waterlogging + 38/31°C; WH3, waterlogging + 42/35°C) for 48 h

Note: The data were mean ± SE (n = 3). Different lowercase letters above the bars indicated significant differences among different treatments ($P < 0.05$).
